# Kimura's disease successively affecting multiple body parts: a case-based literature review

**DOI:** 10.1186/s12886-022-02378-y

**Published:** 2022-04-02

**Authors:** Baodi Yang, Hailan Liao, Minghua Wang, Qiaoyan Long, Huanhuan Zhong, Lin Luo, Zhongmin Liu, Xiaohui Cheng

**Affiliations:** Department of Ophthalmology, Longgang District People’s Hospital of Shenzhen, No.53 Aixin Road, Longgang District, Shenzhen, 518172 China

**Keywords:** Kimura’s disease, Eosinophils, Immunoglobulin E, Orbit, Fossa Cubitalis, Groin

## Abstract

**Background:**

Kimura’s disease is a rare, benign, chronic inflammatory disease that presents as painless, solid masses mainly affecting the deep subcutaneous areas of the head and neck, especially the salivary glands, parotid glands and nearby lymph nodes. It is characterized by elevated peripheral blood eosinophil and Immunoglobulin E (IgE) levels.

**Case presentation:**

A 31-year-old Asian male presented with an orbital space-occupying lesion lasting for 1.5 years. Ten years prior, surgical excision of bilateral fossa cubitalis and groin masses was performed, and the pathological examination showed "lymphoproliferative disease". One year later, masses reappeared near the surgical sites; they grew slowly and shrank after glucocorticoid treatment*.* At this point, admission examinations showed in the peripheral blood an eosinophil proportion of 13.4%, a total IgE level of 26,900.00 IU/mL, prurigo present on the whole body, and multiple palpable masses near the bilateral fossa cubitalis and groin. The left eyeball was exophthalmic. The left elbow mass was excised, and the pathological examination confirmed Kimura’s disease. Oral glucocorticoid therapy is taken and tapering regularly. The eosinophil count returned to normal, the IgE level gradually decreased, the orbital space-occupying lesion and elbow and groin masses shrank significantly, and the whole-body skin prurigo disappeared. Currently, the patient has been in a stable condition for eighteen months.

**Conclusion:**

Our case provides a novel insight that Kimura’s disease should be involved in the differential diagnosis of inflammatory lesion mass of orbit and also supports systemic regular glucocorticoid as a valuable therapy of such condition, but close follow-up and long-term observation are crucial.

## Background

Kimura’s disease (KD) is a rare benign chronic inflammatory disease that manifests as painless, solid masses mainly affecting the deep subcutaneous areas of the head and neck, especially the salivary glands, parotid glands and nearby lymph nodes; the disease can occasionally be accompanied by prurigo and nephrotic syndrome [1–3]. It is characterized by elevated peripheral blood eosinophil and Immunoglobulin E (IgE) levels [4, 5] and it occurs predominantly in young Asian males (20–50 years of age), with a male: female ratio of 3.5:1 to 9:1 [6].

The etiology and pathogenesis of KD are still unclear. It is speculated that continuous antigen stimulation, such as that induced by mosquito bites, parasites, fungi, viruses or other infections, changes the immune regulation of T cells or induces IgE-mediated hypersensitivity, causing the release of eosinophil-stimulating factors, which in turn leads to KD [7]. Considering as an inflammatory process, KD has an excellent prognosis, and no malignant transformation has been reported.

No studies have yet reported the successive occurrence of KD in multiple parts of the body. This report describes the case of an Asian male who developed symptoms successively in different parts of the body, all which sites were unusual, as well as a discussion based on the existing literature.

## Case presentation

A 31-year-old male presented to our institute because of exophthalmos affecting the left eye for 1.5 years; this symptom was accompanied by eye redness, eye swelling and prurigo present on the whole body, but there was no eye pain, diplopia, blurred vision, or other symptoms. The patient used to take small doses of corticosteroids irregularly, which relieved the symptoms, but the symptoms recurred after stopping the drug. The patient stopped taking corticosteroids nearly 1 month before his initial visit. Masses in the cubital fossas and groin had been surgically removed at the local hospital 10 years prior, and the pathology result demonstrated "lymphoproliferative disease". One year later, masses reappeared adjacent to the surgical sites; the masses were slow growing and shrank after the administration of corticosteroids.

The eye examination on admission showed the following: the best-corrected visual acuity of both eyes was 1.0 (international standard visual acuity chart: right eye, -6.50 Spherical Diopter (DS); left eye, -6.00 DS); the non-contact intraocular pressure of both eyes was 17 mmHg; and the exophthalmos values (as measured by a Hertel exophthalmometer) were 14 mm in the right eye and 19 mm in the left eye. The movement of both eyeballs in all directions was normal. Conjunctival congestion and chemosis were observed in the left eye, while no other abnormalities were observed in either eyeball. The general physical examination revealed the following: the masses in the bilateral cubital fossas and groin were palpable, slightly textured, but had distinct borders.

Laboratory examinations were performed on admission. The routine blood tests showed the following results: the proportion of eosinophils in peripheral blood was 13.4% (normal, 0.4% ~ 8%); the absolute eosinophil count was 1.19*10^9/L (normal, 0.02 ~ 0.52*10^9/L); the total IgE level was 26,900.00 IU/mL (normal, ≤ 100); the high-sensitivity C-reactive protein value was 4.5 mg/L (normal, 3); and the IgG4 level was 1.910 g/L (normal, 0.05 ~ 1.540). There were no obvious abnormalities in the levels of vasculitis-related markers (P-ANCA, C-ANCA, MPO-Ab, PR3-Ab, and ACA), anti-extractable nuclear antigen peptide antibody (anti-ENA antibody) or rheumatoid factor, and the results of routine urine and stool tests and liver and kidney function tests were normal. The smear and flow cytometry results of bone marrow aspiration did not show obvious abnormalities related to leukaemia or high-risk myelodysplastic syndrome.

**Fig. 1:**
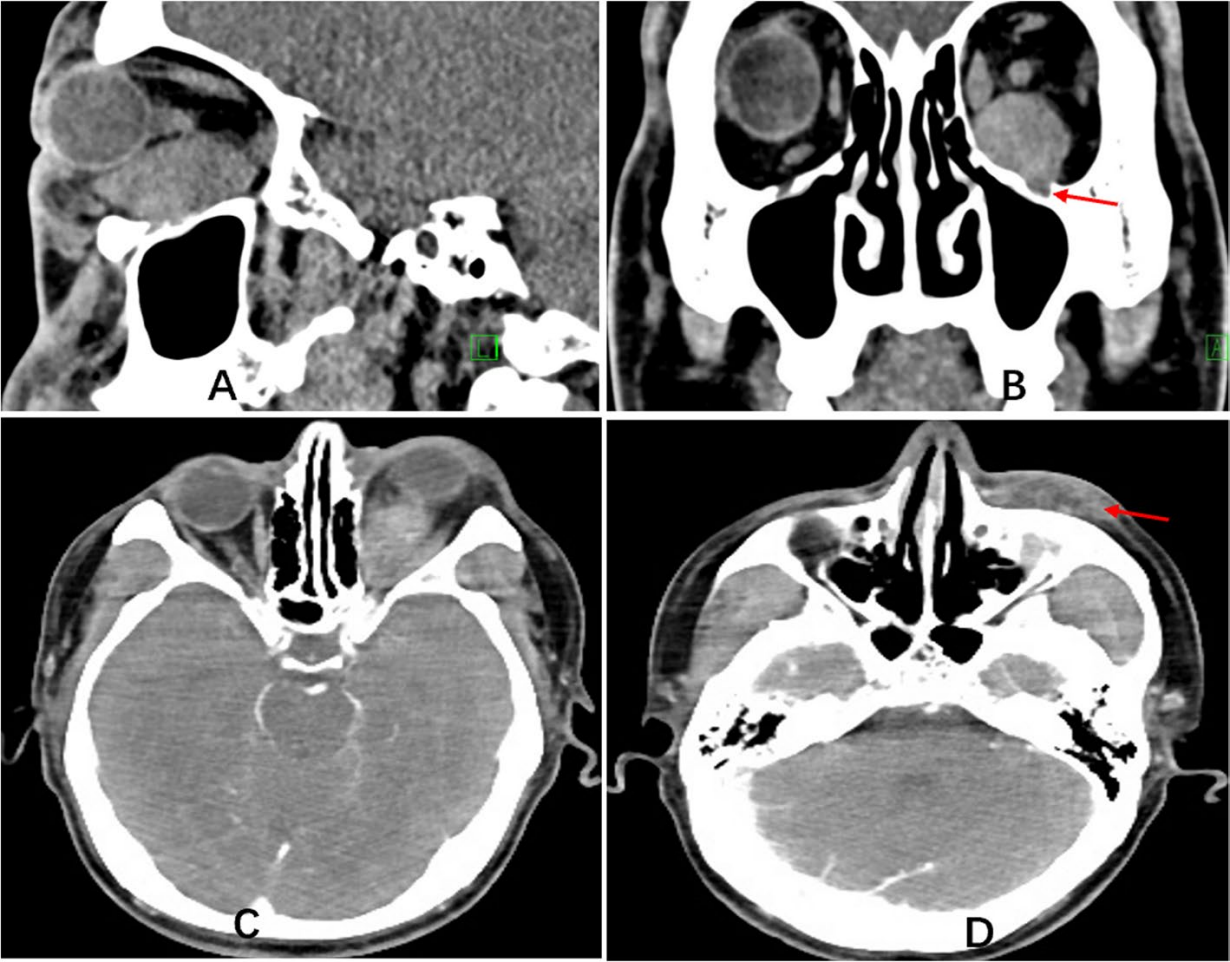
Orbital CT. **A** shows the lesion mass is medium density. The deep part is adhered to the optic nerve (sagittal scan); **B** shows part lesion invades the infraorbital fissure (coronal scan); **C **shows the lesion is adjacent to the medial rectus muscle and is inhomogeneous contrast enhancement (horizontal enhanced scan); **D** reveals the subcutaneous thickening (horizontal enhanced scan)

**Fig. 2:**
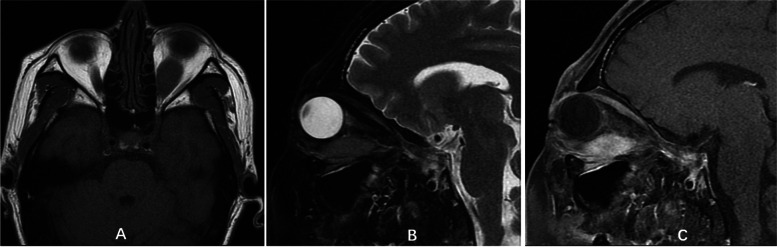
Orbital MRI. T1 weighted image shows the lesion is low signal (**A**, orbital horizontal scan); T2 weighted image shows medium signal (**B**, orbital sagittal scan); The lesion mass is significantly enhanced (**C**, orbital sagittal enhanced scan); Part lesion is adhesion to the inferior rectus muscle and optic nerve (**B** and **C**)

**Fig. 3:**
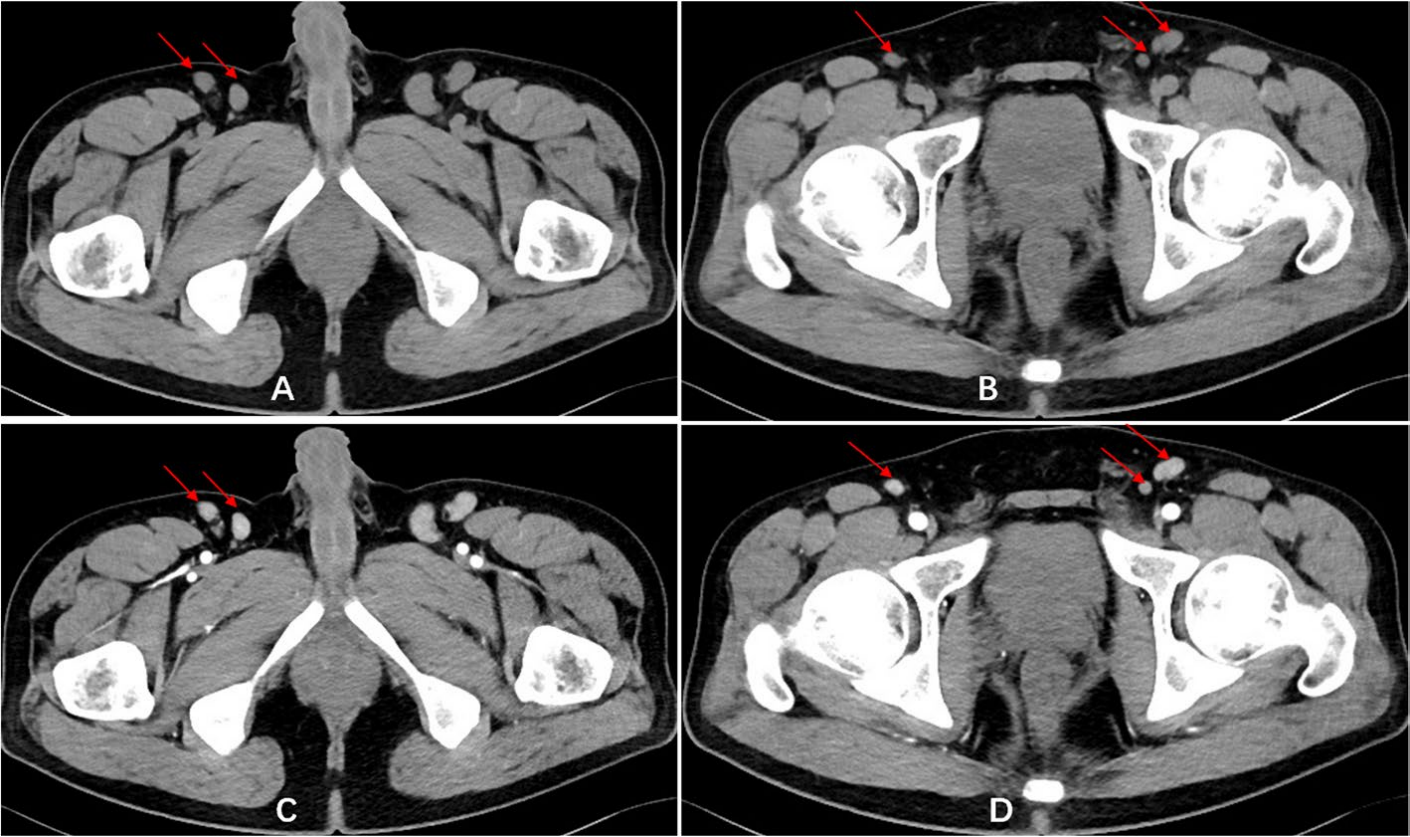
The lower abdomen CT. **A** and **B** reveal multiple nodular masses in the groin on both sides; **C** and **D** reveal inhomogeneous contrast enhancement in the arterial phase

Imaging examinations were performed on admission. Computed tomography (CT) and magnetic resonance imaging (MRI) examinations of the orbit showed a lesion occupying the left orbital subnasal space. The lesion had unclear boundaries, adhered to the medial rectus muscle and inferior rectus muscle, and was locally adjacent to the optic nerve; in addition, there was thickening of the surface subcutaneous tissue. On enhanced orbital MRI, the orbital space-occupying lesion showed significant enhancement. Enhanced orbital CT showed inhomogeneous contrast enhancement of the orbital mass (Fig. 1A-D, Fig. 2A-C). CT of the lower abdomen revealed multiple nodular masses in the groin on both sides, with inhomogeneous contrast enhancement in the arterial phase (Fig. 3A-D). CT of the parotid glands, salivary glands, neck, chest, upper abdomen and kidneys revealed no obvious abnormalities.

**Fig. 4:**
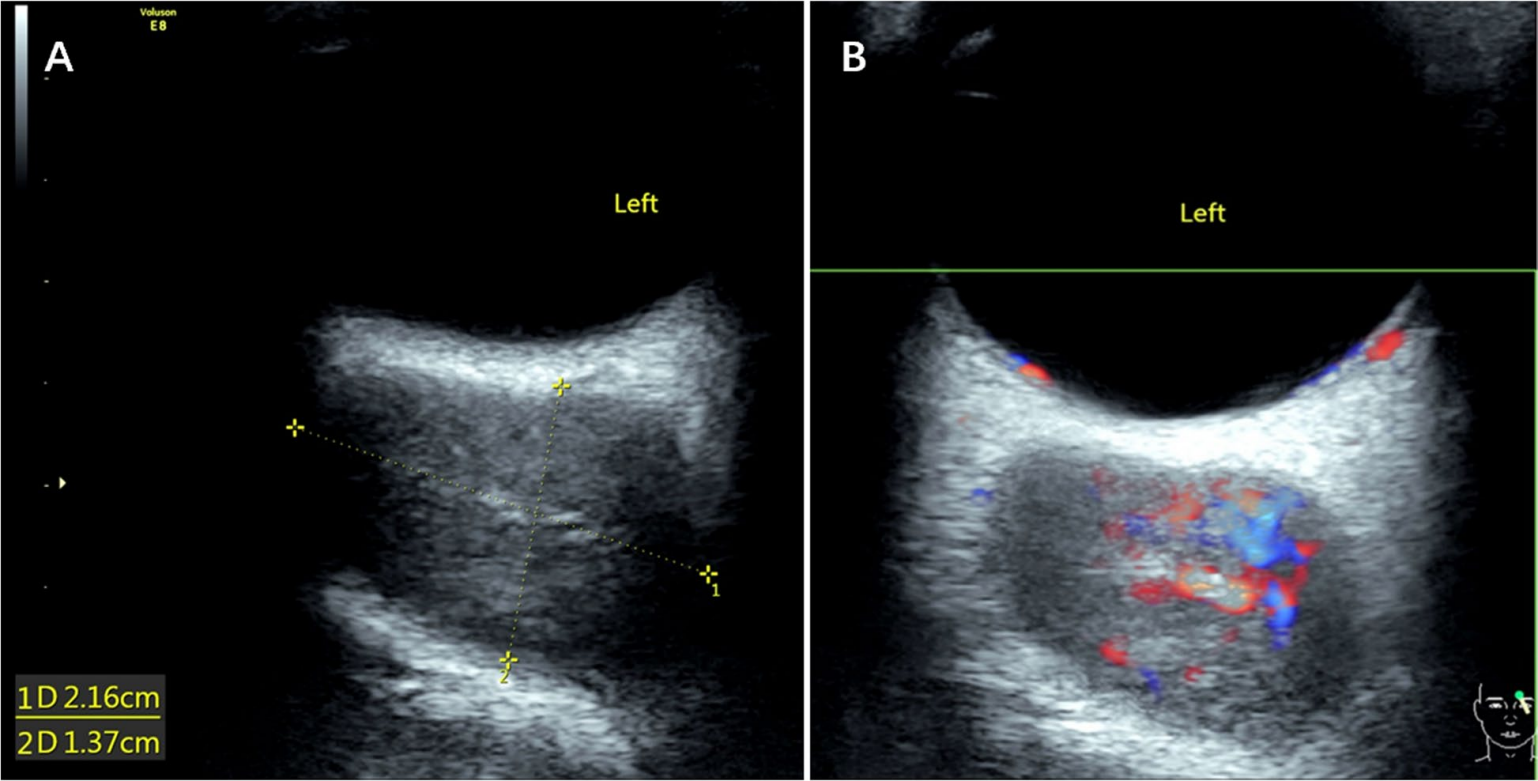
The left orbital colour Doppler ultrasound. The orbital subnasal lesion mass is in the size of 2.16*1.37 cm, and has a heterogeneous hypoechoic-isoechoic appearance; the blood flow is relatively rich

**Fig. 5:**
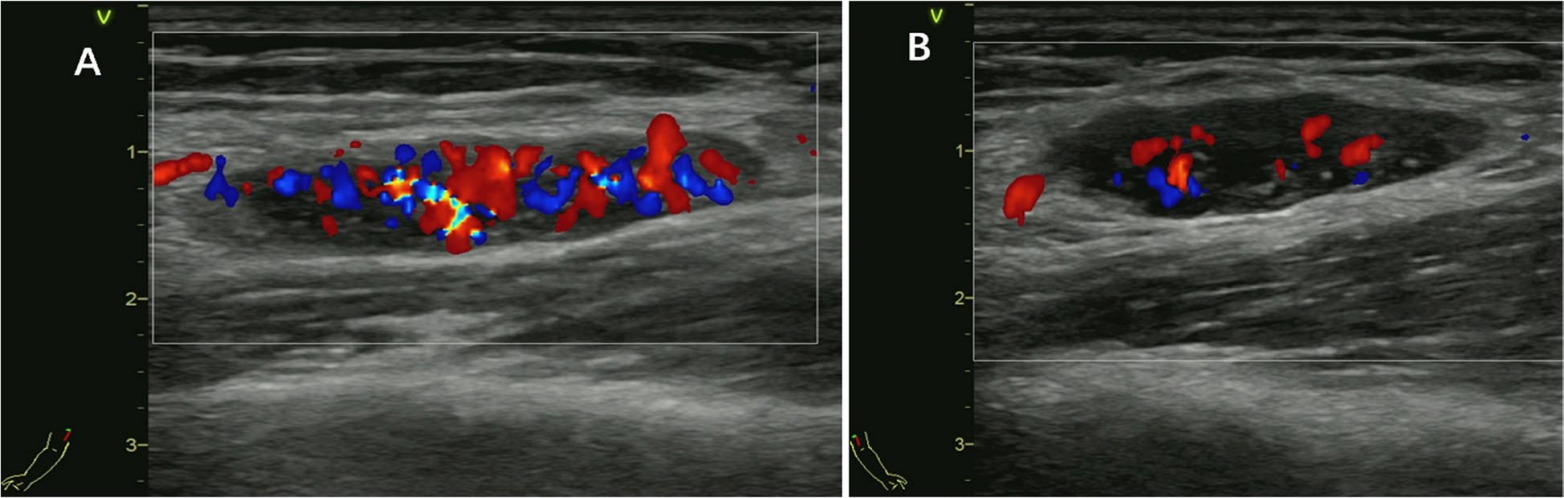
The Colour Doppler ultrasound of bilateral elbow. The bilateral elbow masses are oval in shape and have a heterogeneous hypoechoic-isoechoic appearance; the blood flows are relatively rich. (**A** is the right elbow; **B** is the left elbow)

Colour Doppler ultrasound showed that the left orbital subnasal space-occupying lesion and the bilateral elbow masses were oval in shape and had a heterogeneous hypoechoic-isoechoic appearance; in addition, the signals revealed relatively rich blood flow. (Fig. 4A and B, Fig. 5A and B).

**Fig. 6:**
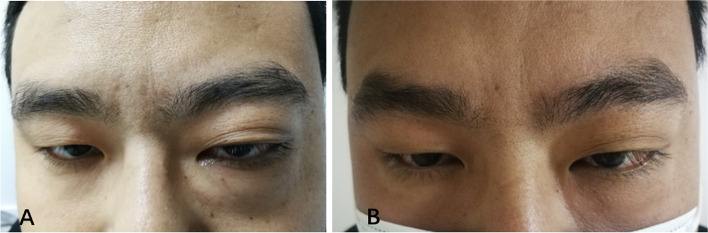
Ocular Appearance. (A) shows the left eyeball protopsis at the time of intravenous drip of methylprednisolone (40 mg) for one day; (B) shows the left eyeball protopsis is significantly alleviative at the time of intravenous drip of methylprednisolone (40 mg) for five days

**Fig. 7:**
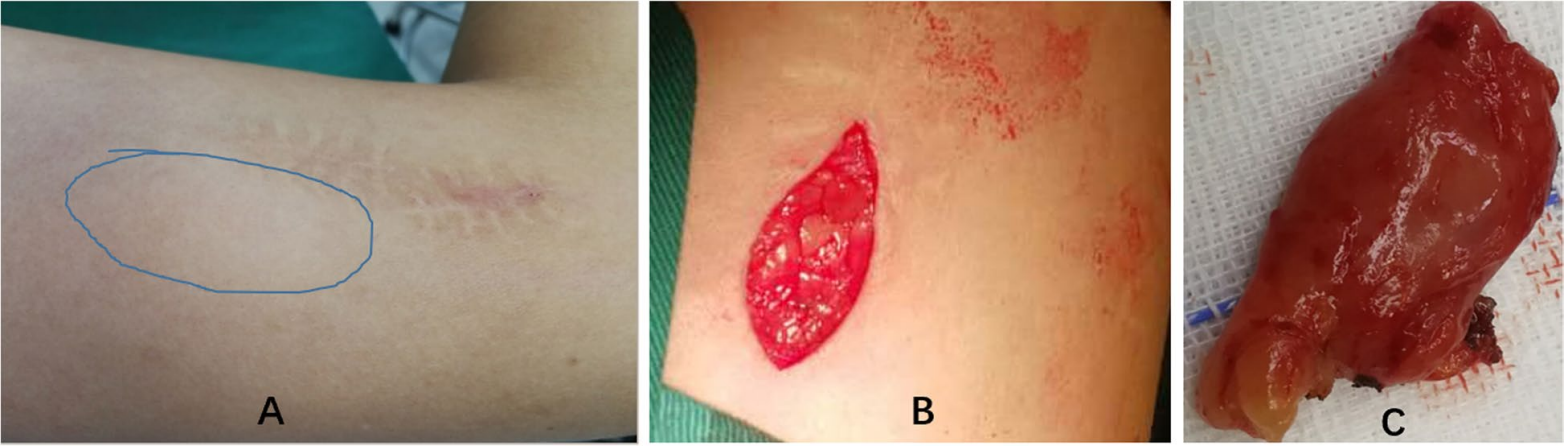
Surgical removal of the left elbow mass. The mass was further for pathological examination

**Fig. 8:**
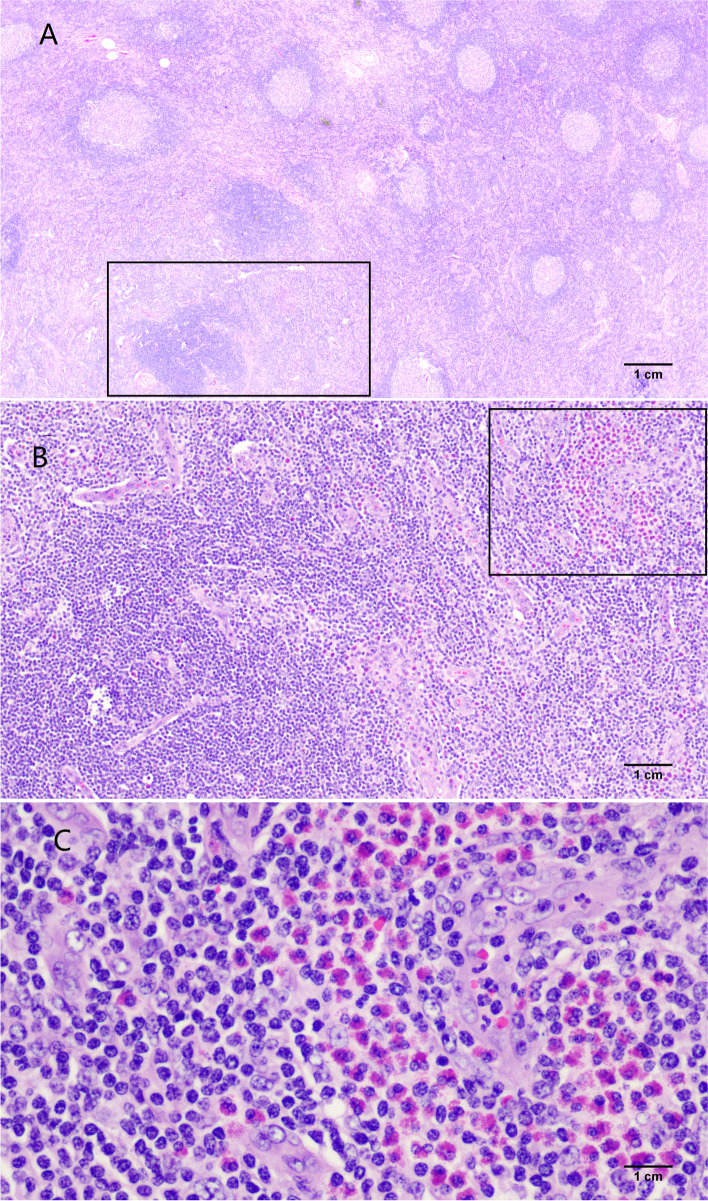
The pathological characteristics of elbow mass. **A** The gross view of pathological sections. In the cortical region, hyperplasia of numerous lymphoid follicles with active germinal centers (× 20); **B** Magnification of the marked region with the rectangle in (**A**). Strong eosinophilic infiltration in the interfollicular area, accompanied by postcapillary venule proliferation with intermingled fibrotic changes. (× 100); **C** Magnification of the marked region with the rectangle in (**B**). Eosinophilic microabscesses (× 400)

On admission, the proptosis, conjunctival congestion and chemosis, and swelling of the patient's left eye were obvious and could not be relieved by the topical administration of tobramycin-dexamethasone eye drops. To alleviate the uncomfortable symptoms, an intravenous drip of methylprednisolone (40 mg) was given for 5 days, which obviously relieved the symptoms (Fig. 6A and B). In addition, the cubital fossas and groin masses were significantly reduced, and the peripheral blood eosinophil proportion (1.4%) and absolute count (0.14*10^9/L) returned to normal. Surgical removal of the left elbow mass was performed at the time of three days with methylprednisolone treatment (Fig. 7A-C). The pathological examination (Olympus Corporation, Japan; Qualcomm Pathology Reporting System) showed hyperplasia of numerous lymphoid follicles with active germinal centres and strong eosinophil infiltration in the interfollicular area (eosinophilic micro-abscesses formation), accompanied by postcapillary venule proliferation with intermingled fibrotic changes (Fig. 8A-C, ImageJ). Based on the patient's medical history, clinical manifestations and pathological characteristics, the patient was diagnosed with KD. Considering that the patient had good visual acuity and positioning and movement of the eye and that the lesion significantly shrank after glucocorticoid treatment, surgical excision was not performed.

After hospital discharge, the patient took oral methylprednisolone tablets (32 mg/d) for 30 days. At the second follow-up visit, the peripheral blood eosinophil proportion (2.5%) and absolute count (0.29*10^9/L) were well within the normal ranges, the total IgE level was decreased to 17,850.00 IU/mL, and the left orbital space-occupying lesion and the fossa cubitalis and groin masses were significantly reduced. In addition, the whole-body skin pruritus and prurigo disappeared. No other complications were observed. The dosage of methylprednisolone was tapering monthly and is currently being maintained by prednisone of 7.5 mg per day. Given the patient's reproductive needs, combined therapy with immunosuppressive agents has not yet been performed. The patient is currently under close follow-up observation and has been in a stable condition for eighteen months.

## Discussion and conclusions

KD was first reported by Kimm in China in 1937, which referred to seven patients characterized by enlarged lymph nodes with pathological eosinophilic infiltration**,** when it was termed as “eosinophilic hyperplastic lymphogranuloma” [8]. In 1948, Kimura et al. reported similar cases and described the details of the pathological characteristics, after which Kimura’s disease was widely recognized [9].

The exact pathogenesis of KD is still unclear. At present, it is believed that triggers, such as bacteria, viruses, and parasites, may change the immune regulation of T cells. Moreover, the immune response mediated by Th2 cells is highly related to KD [10, 11]. The Interleukin-4 (IL-4), IL-5 and IL-13 produced by Th2 cells may be related to the elevated levels of IgE and eosinophil in peripheral blood [12, 13]. In addition, KD seems to have a predilection for Asian young males, so sex hormones and ethnic genetic factors may also contribute to the onset of this disease.

In this case, after administration by methylprednisolone for three days, the peripheral blood eosinophil level returned to normal; while the IgE level decreased slowly, and it was still significantly higher than the normal level. However, all of the lesions on the sites of left orbit, fossa cubitalis and groin significantly shrank, and the skin pruritus, conjunctival congestion and chemosis were relieved completely. Also, considering the pathological findings of eosinophilic infiltration and eosinophilic microabscess formation, the infiltration amounts of eosinophils appear to directly correlate with systemic manifestations of KD [14]; in addition, IgE may play a role as an upstream regulatory factor, stimulating the release of Th2 cytokines, promoting the production of eosinophils, activating inflammatory cells (mast cells, basophils, etc.), and ultimately leading to the clinical manifestations [15]. However, further studies are essential to verify this speculation.

In the diagnosis of KD, we should refer to clinical manifestations, related examinations of laboratory and imaging and the medical history. Moreover, pathological evidence is essential for the definite diagnosis. In this case, the peripheral blood eosinophil and IgE levels were significantly elevated, and lesion masses in the fossa cubitalis and groin had been present for ten years, while the left orbital lesion mass had been emerged for 1.5 years before this admission. According to the pathological findings of the resected fossa cubitalis lesion, KD was diagnosed. After treatment with glucocorticoids, all of the lesion masses significantly shrank. Also, they had similar imaging features. Therefore, there should be sufficient evidence to consider this entity of KD. Furthermore, the orbit was affected later than the other body parts, which is consistent with most of the literature reports [16, 17].

Angiolymphatic proliferative disease with eosinophilic hyperplasia (ALHE) has similar histological features and clinical manifestations as KD, such as eosinophilic granuloma and inflammatory haemangioma nodules. Hence, ALHE and KD were considered as the same disease for a long time. However, ALHE generally occurs between the third and fourth decades of life and appears to have a predilection for women, but no racial differences [18]. In addition, ALHE generally shows no obvious elevation of the peripheral blood IgE or eosinophil level, and lymph nodes are rarely involved. Histologically, vascular proliferation is more obvious in lesions of ALHE than in those of KD, and vascular endothelial cells mostly appear as tall tombstones in shape in ALHE, while in KD vascular endothelial cells are thinner and flatter, and fibrosis is more common [19].

The patient first presented to our institute because of the left eye proptosis and conjunctival congestion and chemosis, and therefore idiopathic orbital pseudotumor needed to be distinguished, which is a benign inflammatory condition and is a diagnosis of exclusion since its aetiology and pathogenesis have been not yet clear. It shows different clinical features according to the special location, degree of inflammation and stages of disease, which may be presented acutely, subacutely, or chronically. Patients may present with ophthalmalgia, diplopia, restriction of extraocular muscle movement, proptosis, congestion and edema of the eyelid or conjunctiva, and even headache. Its pathological features are also diverse, including lymphoproliferative, granulomatous, sclerosing, or eosinophilic infiltrative and vascular inflammatory types [20, 21].

In addition, in this case, the diagnosis is needed to be differentiated from orbital lymphoma, eosinophilic granulomatous polyangiitis, IgG4-related diseases, and parasitic infections, et al. Table 1 summaries the principal characteristics of these diseases, however, the histopathological examination is still the golden standard for diagnosis.

**Table 1:** The different characteristics of Orbital Lymphoma, EGPA and IgG4-RD with Kimura's disease

Diseases	Kimura's disease	Orbital Lymphoma	EGPA	IgG4-RD
Clinical features	An immune-mediated inflammatory disorder; lymphadenopathy; subcutaneous nodules; painless; swelling in the head and neck is common	A malignant tumor arising as clonal expansions of B-lymphocytes, T-lymphocytes, or NK-cells; Lymphadenopathy; B symptoms (fever, night sweats, or weight loss, etc.)	An ANCA associated vasculitis; eosinophil-rich granulomatous; asthma; recurrent pneumonia; paranasal sinus infections; skin lesions (most commonly purpura); Mono- or poly-neuropathy	A fibroinflammatory condition; diffuse/localized swelling in single or multiple organs; non-specific, and overlap with various inflammatory and neoplastic conditions
Laboratory analysis	Peripheral blood eosinophil and IgE levels ↑↑	Depending on histopathological subtype and the clinical stage; anemia, WBC↑are common	Peripheral blood eosinophilia > 10% total WBC; CRP level is median 2.5 ~ 6.6 mg/dl; 30 ~ 47% of patients ANCA ( +); serum IgE and IgG4 level↑	Serum IgG4 > 135 mg/dl
Histopathological features	Hyperplasia of lymphoid follicular; enlargement of germinal center; infiltration or accumulation of eosinophils, even to form “eosinophilic abscess”; hyperplasia of postcapillary and venular with intermingled fibrotic changes	Effacement of lymph node architecture; rich inflammatory background, eosinophils and EBERs may be positive; immunohistochemical features to differentiate between B- and T-cell	Necrotizing small vasculitis accompanied by eosinophil infiltrates; perivascular and extravascular granulomas; palisading granuloma; fibrinoid necrosis in the wall of the vessels; rupture of internal elastic lamina	IgG4 + plasma cell infiltration; IgG4 + /IgG + of > 0.40, or a total of > 50 IgG4 + plasma cells/ high power field; storiform fibrosis; obliterative phlebitis; increased numbers of eosinophils, without eosinophilic granulomas
Available treatments	Glucocorticoids; immunosuppressants; surgery; local low-dose radiotherapy; anti-IgE monoclonal antibody(omalizumab)	Radiotherapy; chemotherapy; Glucocorticoids; surgery; Monoclonal antibodies (rituximab); Stem cell transplant	Glucocorticoids; immunosuppressants; monoclonal antibody: against interleukin-5 (mepolizumab); anti-CD20 (rituximab); Anti-IgE (omalizumab)	Glucocorticoids; immunosuppressants; anti-CD20 monoclonal antibody (rituximab)
References	[11, 22, 23]	[24, 25]	[26, 27]	[28, 29]

*EGPA* eosinophilic granulomatosis with polyangiitis, *IgG4-RD* IgG4-related disease, *ANCA* antineutrophil cytoplasmic antibody, *WBC* white blood cells, *CRP* c-reactive protein, *EBERs* EBV-encoded RNAs

According to the available literature, effective treatments for KD include the systemic administration of glucocorticoids or immunosuppressive agents (e.g., cyclosporine, cyclophosphamide, imatinib, or methotrexate), the surgical resection of local lesions, and local low-dose radiotherapy or biological immunotherapy, such as with an anti-IgE antibody (omalizumab) [11, 14, 30]. However, KD has a high recurrence rate, which reaches up to 62% [31]. There is not yet an optimal therapeutic strategy. Ma et al. reported a case of KD affecting the bilateral upper eyelids and parotid glands in which the patient was given oral prednisone at 40 mg combined with intravenous methotrexate at 15 mg every week for 2 months. The clinical symptoms were completely relieved, and no recurrence was observed over a follow-up period of 2 years [32]. Nonaka et al. reported that the subcutaneous injection of anti-IgE antibody (omalizumab at 300 mg per day) once every two weeks for a total of 8 treatment cycles could effectively treat Kimura’s disease without any adverse reactions [15]. A systematic review on the role of radiotherapy in KD provides us a comprehensive and up-to-date perception of the potential benefits of radiotherapy, which may play a role either as an exclusive or an adjuvant treatment modality, however, additional investigations are indispensable in the future [33].

In this case, the borders of the left orbital lesion mass were vague, which was adherent to the extraocular muscles and optic nerve and partially invaded the inferior orbital fissure. And there were abundant blood vessels in the orbital lesion mass. Complete surgical excision was considered very difficult because there were high risks of not only damaging the extraocular muscles or optic nerve but also bleeding. The patient's visual function has not yet been affected, and the lesion mass significantly shrank after glucocorticoid treatment. Moreover, no adverse drug reactions were observed after glucocorticoid treatment. Therefore, to avoid surgical complications, orbital surgery was not performed. As the orbital lesion mass was close to the optic nerve and nasopharyngeal region, radiotherapy was not administered to prevent damage to the optic nerve or avoid the induction of nasopharyngeal carcinoma. Immunosuppressive agents have still not been administered because of the patient’s reproductive wishes. However, close follow-up and observation are being performed to ascertain the minimum effective dose of glucocorticoids and determine whether additional surgical resection or immunosuppressive therapy is required.

As the disease progresses, the blood vessels in the lesion mass may gradually atrophy, and fibrosis may develop. As such, KD displays a benign pathological progression that is likely to be relieved spontaneously [34, 35].

Our case provides a novel insight that Kimura’s disease should be involved in the differential diagnosis of inflammatory lesion mass of orbit and also supports systemic regular glucocorticoid as a valuable therapy of such condition, but close follow-up and long-term observation are crucial. In addition, it also serves as a reminder that ophthalmologists should have a complete systematic evaluation in order to make prompt and accurate diagnosis, sparing the patient any unnecessary or potentially harmful medical procedures.

## Data Availability

The datasets used and analysed during the current study are available from the corresponding author on reasonable request.

## Abbreviations

KD: Kimura’s disease
IgE: Immunoglobulin E
DS: Spherical diopter
CT: Computed Tomography
MRI: Magnetic Resonance Imaging
IL: Interleukin
ALHE: Angiolymphatic proliferative disease with eosinophilic hyperplasia
